# PRP-Fibrin Glue for Pain Reduction and Rapid Healing in Tonsillectomy

**DOI:** 10.22038/IJORL.2022.40429.2532

**Published:** 2022-11

**Authors:** Navid Nourizadeh, Nooshin Sedaghat Sharifi, Mehdi Bakhshaee, Shirin Sadat Ghiasi, Bashir Rasoulian, Daryoush Hamidi Alamdari

**Affiliations:** 1 *Sinus and Surgical Endoscopic Research Center, Mashhad University of Medical Sciences, Mashhad, Iran.*; 2 * Faculty of Medicine, Mashhad University of Medical Sciences, Mashhad, Iran*; 3 *Department of Otorhinolaryngology, Head and Neck Surgery, Sinus and Surgical Endoscopic Research Center, Mashhad University of Medical Sciences, Mashhad, Iran.*; 4 *Surgical Oncology Research Center, Mashhad University of Medical Sciences, Mashhad, Iran*

**Keywords:** Fibrin glue, Healing, Platelet-Rich-Plasma, Pain, Tonsillectomy

## Abstract

**Introduction:**

Many ongoing challenges have been applied to reduce the considerable postoperative pain and increase wound healing after tonsillectomy, but they are still not optimally managed. This study applied autologous platelet-rich plasma (PRP) & platelet-rich fibrin glue (PRFG) to reduce pain and increase wound healing.

**Materials and Methods:**

PRP & PRFG were prepared from 26 patients’ blood. At the end of the tonsillectomy, one tonsillar bed was selected randomly, PRP was injected, PRFG was applied topically on the bed wound, and the other sites were left untreated. The treated and untreated tonsillar beds were compared for pain and wound healing the next day, 3rd day, 6th day, 9th day, and 15th day.

**Results:**

There were no complications during and after the injection. The mean age was 24.76 ±5.54 years. In the treated beds in comparison to untreated beds, pain decreased marginally in 1st day (intervention:4.5±2.54, control:5.53±2.94, P-value=0.18) and 3rd day (intervention:3.92±2.96, control:4.8±2.82, P-value=0.276), and significantly in 6th day (intervention:2.3±2.46, control:3.92±2.6, P-value=0.026), 9th day (intervention:1.26±1.48, control:2.76±2.4, P-value=0.009) and 15th day (intervention:0.73±1.07, control:1.84±2.36, P-value=0.08) after surgery. Healing did not change in 1st day (P-value=1), changed marginally in 3rd day (P-value=0.2), and increased significantly in 6th day (P-value=0.001), 9th day (P-value=0.006), and 15th day (P-value=0.004) after surgery.

**Conclusions:**

Autologous PRP injection & PRFG application offer an effective, safe, and non-invasive method for reducing pain and increasing wound healing after tonsillectomy.

## Introduction

In otolaryngology, one of the most common surgical operations is a tonsillectomy, and severe pain and wound healing remain major problems that affect patients profoundly after surgery. Pain causes a loss of time to return to a regular diet and normal activity (1). Many challenges have been done to reduce severe pain and increase wound healing, but still, there is no perfect analgesic strategy for this approach. Pain medications given to patients can lead to nausea, vomiting, and constipation (2). Also, there are concerns about those children unwilling to take in fluids and pain medications. One study reported that over 40% of children were given two or fewer doses of postoperative pain medication one day after tonsillectomy, and over 60% were given two or fewer doses on postoperative day 2 (3). 

There is a safety concern about medications. It is reported that no medication is effective if not properly prescribed. Precise instructions must be given to caregivers to make straightforward, safe, correct, and timely prescriptions of pain medications (4). Also, in tonsillectomy, an increasing number of mortalities related to codeine, one of the most used drugs for treating postoperative pain in children, warn physicians to develop a new medical approach to control pain (5). We report a clinical trial done for 26 patients aiming to assess the effect of autologous platelet-rich plasma (PRP) & platelet-rich fibrin glue (PRFG) on reducing pain and increasing wound healing after tonsillectomy. A literature review showed that the combination of PRP & PRFG had been used in this management for the first time.

## Materials and Methods

Patients

Twenty-six patients were included in the study at Imam Reza academic hospital. The patients’ characteristics are presented in Table 1.

**Table 1:** *The characteristics of patients*

**Variable**		
Sex	Female No. (%)	14 (54)
	Male No. (%)	12 (46)
Age (Mean ± SD) years (Min, Max) years		24.76±5.54 (14, 38)

According to the Declaration of Helsinki principles and Good Clinical Practice standards, the study was done. The Human Research Ethics Committee of Mashhad University of Medical Sciences approved the study protocol and informed-consent form. All patients signed informed consent. Inclusion criteria: Age 14 years and older, recurrent tonsillitis or obstructive tonsillar hypertrophy requiring tonsillectomy. Exclusion Criteria: history of peritonsillar abscess, concurrent illness with impairment of wound healing (diabetes, vascular disease, end-stage renal disease), pregnancy, breastfeeding, asthma, epilepsy, inability to comply with the protocol conditions.

Autologous PRP & PRFG preparation

1. Two days before the operation, 60ml peripheral blood was taken in 9ml clinical grade citrate buffer.
2. 4ml PRP was prepared by first centrifugation at 2000g for 2 Minutes and then second centrifugation at 4000g for 8 Minutes, and the supernatant plasma was separated and left 4ml PRP (6). Two ml was kept as PRP to inject the tonsillar bed, and the other 2 ml of PRP was mixed with 2ml fibrinogen concentrate (following step3) to make the platelet-rich fibrinogen plasma (PRFP) [final volume: 4ml].
3. The fibrinogen was prepared from the above plasma (step2) by cryoprecipitate method (7). Following a -70ºC freeze and a 4ºC thaw, plasma was centrifuged at 6500g for 5 Minutes. The precipitated fibrinogen was separated from the supernatant plasma to a final volume of 2ml.
4. Thrombin preparation: to 10ml separated plasma from step 3, 10% calcium gluconate was added.
5. The clot was formed after 30 Minutes, it was shaken vigorously and centrifuged at 3500g for 3 Minutes, and 1ml supernatant plasma was separated, which contains thrombin.
6. Platelet-rich fibrin glue preparation (PRFG): 4ml PRFP was mixed with 1ml thrombin in the operation room.

***PRP Injection and PRFG application***

Before tonsillectomy, one gram of Cephazolin was given intravenously. The blunt dissection technique was used for tonsillectomy. One tonsillar bed was selected randomly, under general anesthesia, 2ml of PRP was injected in, PRFG was applied topically on the bed wound, and the other sites were left untreated. The treated and untreated tonsillar beds were compared for pain and wound healing the next day and every three days until the 15th. The patients were discharged the next day after the operation. 

***Pain and wound healing evaluation***

**Fig 1:**
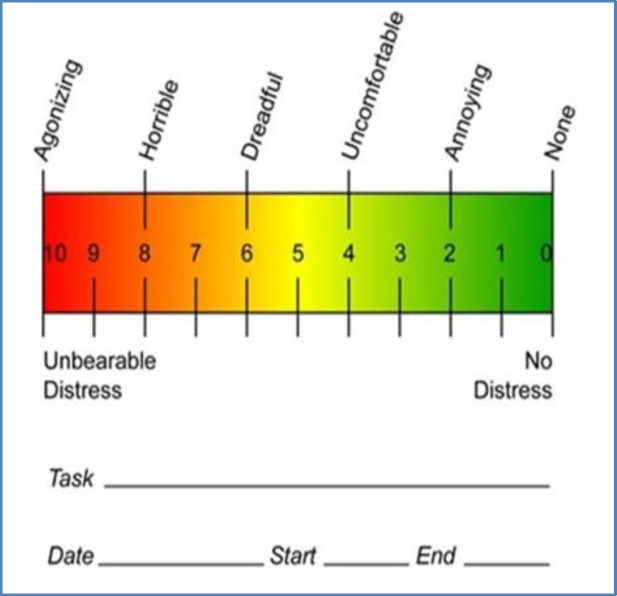
Visual linear analog scale (VAS) (0-10 NUMERIC PAIN DISTRESS SCALE)

A visual linear analog scale was used for measuring pain severity (Figure 1). The patient & the second physician were blinded to the treated side to eliminate bias. A wound healing scale was defined according to our experience. Wound healing was determined based on the amount of a tonsillar bed covered with a fibrinous layer (Table 2).

**Table 2:** Classification of healing of tonsillar beds

**Coverage rate of tonsillar beds with a fibrin layer**	**Healing Rate**
Fibrin layer covers 2/3 up to the total tonsillar bed	little healed
Fibrin layer covers 1/3 up to 2/3 tonsillar bed	partial healed
Fibrin layer covers 1/3 of the tonsillar bed to the complete resolution	total healed

Statistical analysis

The Statistical Package for Social Sciences (SPSS version 11.5) was applied for data analysis. Mean and standard deviation was measured for all variables. Values are reported as mean ± SD. A level of P-value < 0.05 was considered statistically significant.

## Results

**Fig 2:**
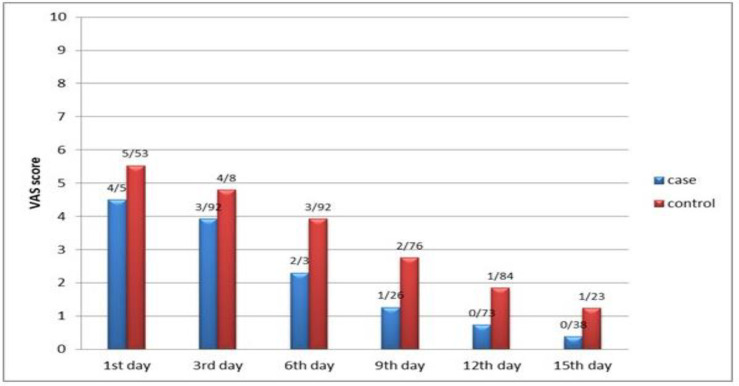
Chart of median pain score in different days (VAS): intervention tonsillar bed (case) and control tonsillar bed

There were no complications during and after the injection. The mean age was 24.76 ±5.54 years. In the treated beds in comparison to untreated beds, pain decreased marginally in 1^st ^day (intervention: 4.5±2.54, control: 5.53±2.94, p-value: 0.18) and 3^rd ^day (intervention: 3.92±2.96, control: 4.8±2.82, p-value: 0.276) and significantly in 6^th ^day (intervention: 2.3±2.46, control: 3.92±2.6, p-value: 0.026), 9^th^ day (intervention: 1.26±1.48, control: 2.76±2.4, p-value: 0.009), and 15^th^ day (intervention: 0.73±1.07, control: 1.84±2.36, p-value: 0.08) after surgery (Figure 2). Healing did not change on 1^st ^day (intervention: 26 little healed, 0 partial healed, 0 complete healed, control: 26 little healed, 0 partial healed, 0 complete healed, p-value: 1). 

It changed marginally on 3^rd ^day (intervention: 24 little healed, 2 partial healed, 0 complete healed, control: 26 little healed, 0 partial healed, 0 complete healed, p-value:0.2). Moreover, healing increased significantly in 6^th ^day (intervention: 6 little healed, 19 partial healed, 1 complete healed, control: 21 little healed, 5 partial healed, 0 complete healed, p-value:0.001), 9^th^ day (intervention: 0 little healed, 23 partial healed, 3 complete healed, control: 7 little healed, 18 partial healed, 1 complete healed, p-value:0.006), and 15^th^ day (intervention: 0 little healed, 4 partial healed, 22 complete healed, control: 0 little healed, 14 partial healed, 12 complete healed, p-value:0.004) after surgery (Table 3).

**Table 3:** Healing in intervention tonsillar bed (I) and control (C) tonsillar bed

**Day**	**1st**		**3th**		**6th**		**9th**		**15th**	
	I	C	I	C	I	C	I	C	I	C
little healed	26	26	24	26	6	21	0	7	0	0
partial healed	0	0	2	0	19	5	23	18	4	14
complete healed	0	0	0	0	1	0	3	1	22	12
p-value	1		0.2		0.001		0.006		0.004	

## Discussion

This study shows that PRP and PRFG can decrease significantly and safely the pain score and increase wound healing after tonsillectomy. In this study, the outstanding success rate of pain reduction and wound healing is related to the usage of platelets and FG, which are the key contributing factors in regenerative tissue.

After surgery, severe pain decreases oral intake, causes dehydration, or latency in recovery. The most used drug for lowering post-tonsillectomy pain is acetaminophen, but it cannot completely relieve pain. Also, other drugs such as nonsteroidal anti-inflammatory drugs, opiates, injectable steroids, topical anesthetizing sprays, or sucralfate are applied to reduce post-tonsillectomy pain. However, these agents’ efficacy and side effects demand more investigations to discover post-tonsillectomy pain relieving. One study reported that following tonsillectomy, the oral usage of honey can decrease postoperative pain in pediatric patients and may significantly reduce the need for analgesics (8,9).

The best wound healing necessitates a well-orchestrated integration of the complex biological and molecular actions of cell migration, proliferation, extracellular matrix deposition, and remodeling (10), in which regenerative growth factors and a strong scaffold have critical roles in starting and continuing these events. PRP provides the optimum local concentration of growth and bioactive factors for wound healing in the α-granules and the dense granules, respectively, in platelets. The α-granules have growth factors such as transforming growth factor–β, platelet-derived growth factor, insulin-like growth factor, fibroblast growth factor, epidermal growth factor, vascular endothelial growth factor, and endothelial cell growth factor. These growth factors have main roles in cell proliferation, chemotaxis, cell differentiation, and angiogenesis. 

Dense granules have bioactive factors such as serotonin, histamine, dopamine, calcium, and adenosine. Histamine and serotonin enhance capillary permeability, allowing inflammatory cells to enter the wound site more and stimulating macrophages (11).

In sum, the bioactive factors have main roles in the healing events by modulating the recruitment, duplication, activation, and differentiation (12). 

PRP has the best effect when its concentration is 4-5 times more than the baseline in the blood (one million platelets per microliter). Lesser concentrations cannot improve wound healing, and greater concentrations fail to increase wound healing. Also, the antimicrobial activity of PRP has been proven against *Escherichia coli*, *Staphylococcus aureus*, *Candida albicans*, and *Cryptococcus neoformans* (13).

FG is a topical biological sealant in which thrombin splits off monomers of fibrinopeptide A and B from the fibrinogen chain, which polymerizes to create a strong fibrin clot at the application site is ten times stronger than the physiological clot. This clot creates an important provisional extracellular matrix and actively employs cells to start fibrin-mediated responses, such as cell adhesion, migration, and proliferation (14). 

Fibrin clot promotes wound healing by creating a temporary matrix, stimulating the local proliferation of fibroblasts and collagen synthesis and following replacement by connective tissue, new blood vessel formation, and stopping fibrosis (15, 16). In this study, fibrin clot has two main roles: 1) delivering of growth factors to stimulate wound healing and 2) providing a temporary strong scaffold. In this study, the application of autologous FG instead of allogenic FG is the additional value of this method which reduces the expenses and stops the transition of blood-born diseases via the commercial FG.

## Conclusion

 Autologous PRP injection & PRFG application onto the wound offer a safe, effective and non-invasive method for reducing pain and increasing wound healing after tonsillectomy. 
